# Mindset Moderates Healthcare Providers' Longitudinal Performance in a Digital Neonatal Resuscitation Simulator

**DOI:** 10.3389/fped.2020.594690

**Published:** 2021-02-16

**Authors:** Chang Lu, Simran K. Ghoman, Maria Cutumisu, Georg M. Schmölzer

**Affiliations:** ^1^Centre for Research in Applied Measurement and Evaluation, University of Alberta, Edmonton, AB, Canada; ^2^Department of Educational Psychology, Faculty of Education, University of Alberta, Edmonton, AB, Canada; ^3^Centre for the Studies of Asphyxia and Resuscitation, Neonatal Research Unit, Royal Alexandra Hospital, Edmonton, AB, Canada; ^4^Department of Pediatrics, Faculty of Medicine and Dentistry, University of Alberta, Edmonton, AB, Canada; ^5^Department of Computing Science, Faculty of Science, University of Alberta, Edmonton, AB, Canada

**Keywords:** digital simulation, computer-based game simulation, serious games, performance, mindset, simulation based medical education, neonatal resuscitation program, neonatal resuscitation

## Abstract

**Background:** Simulation education can benefit healthcare providers (HCPs) by providing opportunities to practice complex neonatal-resuscitation tasks in low-stake environments. To our knowledge, no study investigated the role of growth mindset on longitudinal performance on neonatal resuscitation before and after simulation-based training.

**Objectives:** This study examines whether 1) the RETAIN digital/table-top simulators facilitate HCPs' neonatal resuscitation knowledge gain, retention, and transfer and 2) growth mindset moderates HCPs' longitudinal performance in neonatal resuscitation.

**Methods:** Participants were *n* = 50 HCPs in a tertiary perinatal center in Edmonton, Canada. This longitudinal study was conducted in three stages including 1) a pretest and a mindset survey, immediately followed by a posttest using the RETAIN digital simulator from April to August 2019; 2) a 2-month delayed posttest using the same RETAIN neonatal resuscitation digital simulator from June to October 2019; and 3) a 5-month delayed posttest using the low-fidelity table-top neonatal resuscitation digital simulator from September 2019 to January 2020. Three General Linear Mixed Model (GLMM) repeated-measure analyses investigated HCPs' performance on neonatal resuscitation over time and the moderating effect of growth mindset on the association between test time points and task performance.

**Results:** Compared with their pretest performance, HCPs effectively improved their neonatal resuscitation knowledge after the RETAIN digital simulation-based training on the immediate posttest (*Est* = 1.88, *p* < 0.05), retained their knowledge on the 2-month delayed posttest (*Est* = 1.36, *p* < 0.05), and transferred their knowledge to the table-top simulator after 5 months (*Est* = 2.01, *p* < 0.05). Although growth mindset did not moderate the performance gain from the pretest to the immediate posttest, it moderated the relationship between HCPs' pretest and long-term knowledge retention (i.e., the interaction effect of mindset and the 2-month posttest was significant: *Est* = 0.97, *p* < 0.05). The more they endorsed a growth mindset, the better the HCPs performed on the posttest, but only when they were tested after 2 months.

**Conclusions:** Digital simulators for neonatal resuscitation training can effectively facilitate HCPs' knowledge gain, maintenance, and transfer. Besides, growth mindset shows a positive moderating effect on the longitudinal performance improvement in simulation-based training. Future research can be conducted to implement growth-mindset interventions promoting more effective delivery of technology-enhanced, simulation-based training and assessment.

## Introduction

Approximately 10% of newborns around the world need basic medical assistance to make the fetal-to-neonatal transition, with 1% of newborns requiring more complex advanced resuscitative interventions including oxygenation and ventilation, chest compressions, volume expansion, vascular access, and cardiac medications (1–3). The delivery room exposes healthcare providers (HCP) to a challenging, dynamic, and high-pressure setting, where HCPs must coordinately apply meta-cognitive and cognitive skills to analyze and integrate information, communicate with coworkers or patients, make decisions, and perform precise operations to successfully complete neonatal resuscitation tasks (4–7). The low occurrence of high-stake neonatal resuscitation situations and the stressful environment in the delivery room are likely to induce human errors, which may cause fatal consequences for newborns (8–10). Hence, frequent simulation-based training is recommended by the Neonatal Resuscitation Program (NRP) to refresh neonatal resuscitative knowledge and skills every 2 years (11).

Novel technologies such as digital simulators make simulation-based training and assessments accessible for HCPs, so that they can practice and self-evaluate the high-acuity, low-occurrence (HALO) neonatal resuscitation tasks in user-friendly, low-stake environments (12–17). Through recurrent training and assessment of the HALO scenarios, HCPs can effectively maintain and improve their performance in real-life clinical neonatal resuscitation settings (18–20). Previous studies have been conducted to examine factors that influence the effectiveness of simulation-based training. Findings revealed that the performance on simulation-based neonatal resuscitation tasks is determined by different components, such as emotional factors (21–23), socio-environmental factors (24), and organizational factors (24, 25). However, few studies examined the effect of mindset on the knowledge acquisition and retention in simulation-based neonatal resuscitation.

Mindset, an implicit theory of intelligence (26), is defined as the set of beliefs on individuals' learning motivation and ability and it is dichotomized into growth mindset and fixed mindset. Growth mindset refers to the belief that individuals' ability is not inherent and it can be developed and improved through efforts and hard work. On the other hand, a fixed mindset is defined as the belief that ability and intelligence are immutable human traits and that learning cannot improve perceived ability. Previous studies revealed mixed results on how growth mindset influences performance (27–34). For example, Yeager et al. (33) conducted a national online growth mindset intervention for students in secondary education in the United States, which revealed that growth mindset improved lower-achieving students' academic performance (*n* = 5,650 students, *k* = 65 schools) and increased enrolment in advanced mathematics courses. On the other hand, Sisk et al. (30) conducted two rounds of meta-analyses on the effectiveness of growth mindset interventions on increasing students' academic achievement around the world. In the first round (*k* = 273, *N* = 365,915), they examined the relationship between mindset and academic performance moderated by academic risk status, student developmental stage, socioeconomic status, and type of academic achievement measure. In the second meta-analysis (*k* = 43, *N* = 57,155), they examined the effectiveness of mindset interventions on academic achievement moderated by student factors, control and intervention method factors, and academic-achievement-measure-related factors. They found that the effects of mindset and mindset interventions were weak in both meta-analyses.

While many studies have examined the impact of mindset on learning outcomes of children or students in higher-education institutions, few have discussed the influence of mindset on the training outcomes of highly skilled professionals. To our knowledge, no study has examined the effect of growth mindset on the longitudinal performance trajectory of individuals' performance in neonatal resuscitation. Therefore, this study aims to examine whether technology (digital/table-top simulators) could effectively help HCPs learn neonatal resuscitation knowledge, and how growth mindset moderates longitudinal performance in neonatal resuscitation. We hypothesized that HCPs with higher endorsement of growth mindset would demonstrate better performance on the simulation-based assessments.

## Methods

### Study Setup

The present study was conducted at the Centre for the Studies of Asphyxia and Resuscitation using the RETAIN (REsuscitation TrAINing for healthcare professionals) simulator. RETAIN was designed to simulate real-life scenarios in the delivery room, so that HCPs can practice their knowledge on neonatal resuscitation (14).

### Participants

Fifty HCPs (44 females and 6 males), including 27 registered nurses, 14 respiratory therapists, 6 doctors, and 3 neonatal nurse practitioners, were recruited from the Neonatal Intensive Care Unit at the Royal Alexandra Hospital, Edmonton, Canada. The present study was approved by the Human Research Ethics Board at the University of Alberta (Pro00064234). At the study site, healthcare providers working in the Neonatal Intensive Care Unit (NICU), our participant population, take the NRP course once every 2 years, which includes a few hours of in-person small-team simulation with a trained instructor. The time (in months) since participants' last NRP course was reported in the study. Informed consent was obtained from all participants prior to their participation.

### Study Design

Recruited participants were first presented with a tutorial in the RETAIN digital simulator, based on a neonatal resuscitation scenario of an apneic 24-week infant. Then, participants completed a pretest using the RETAIN digital simulator, based on a neonatal resuscitation scenario of an apneic infant with fetal bradycardia. The pretest resuscitation task scenario requires participants to perform a series of actions, including the following eight steps: (1) prepare for the delivery; (2) assist with the baby being born; (3) complete initial assessment; (4) complete basic interventions; (5) initiate ventilation; (6) establish an alternative airway and confirm correct tube placement; (7) initiate cardiovascular interventions; and (8) postresuscitation. More details on each step can be found in Appendix 1.1.

Two practice scenarios were provided to the participants after using the RETAIN digital simulator. The duration of this training was ~20–30 min, with a 1:1 ratio between participant and research supervisor. Participants received no additional simulation training (i.e., in a skills lab with a manikin). A posttest was administered immediately after the training using the same simulated scenario as in the pretest. Then, participants completed a five-point Likert scale (1: “*Strongly Disagree*,” 2: “*Disagree*,” 3: “*Neutral*,” 4: “*Agree*,” and 5: “*Strongly Agree*”) survey to assess their mindset. Two items were designed to assess their growth mindset: (1) *You can always change how good you are at your job* and (2) *You can get better at your job with practice*. Two items aimed to measure their fixed mindset: (1) *You can't really do much to change how good you are at your job* and (2) *You can learn new things, but you cannot really change how good you are at your job*.

Two delayed posttests were administered after 2 and 5 months to ensure that there was enough distance between the pretests and posttests to test long-term memory and transfer instead of working memory. The posttest after 2 months employed identical scenarios to the pretest and immediate posttest using a digital simulator, whereas the posttest after 5 months included more difficult scenarios of an apneic infant with thick meconium using a table-top simulator. The posttest after 5 months was a knowledge-transfer test, where a different task was used to ascertain whether participants learned the concepts more deeply. Specifically, scenarios in the posttest after 5 months required participants to complete the following ten steps: (1) prepare for the delivery; (2) assist with the baby being born; (3) complete initial assessment; (4) complete basic interventions; (5) initiate ventilation; (6) establish an alternative airway and confirm correct tube placement; (7) initiate cardiovascular interventions; (8) establish vascular access; (9) administer medication; and (10) postresuscitation. More details of each step can be found in Appendix 1.2.

### Measures

#### Outcome Variable

Participants' raw scores of the four simulation-based assessments were used as the outcome variable. The pretest measured participants' baseline neonatal resuscitation knowledge. The immediate posttest revealed their knowledge gains after the simulation-based training. The posttest after 2 months examined whether participants retained the neonatal resuscitation knowledge using the same assessment medium. Moreover, the posttest after 5 months revealed whether they transferred the knowledge to a new medium.

On the pretest, the immediate posttest, and the posttest after 2 months, participants' actions including keystrokes and mouse clicks were automatically recorded by the digital simulator as. txt files. On the posttest after 5 months, performances based on the table-top simulator were recorded by a supervising researcher using a checklist. One researcher scored the performance of all HCPs on the 5-month tabletop simulation. The researcher was not associated with the hospital and was not involved in the participants' employment or professional evaluation. Binary scores were assigned to the task performances in the digital and table-top simulators guided by the 7th edition NRP guidelines, where a score of 1 referred to 100% adherence to the guidelines, and a score of 0 referred to <100% adherence to NRP guidelines. More specifically, participants' performance was scored with either pass or fail for each scenario (binary score with a maximum of 1 and a minimum of 0). Pass represented 100% adherence to NRP guidelines, whereas Fail represented <100% adherence to NRP guidelines (7th edition, 2015 NRP). While the scenarios were scored as pass or fail, the sequence was flexible enough to accommodate clinical relevance. Appendix 1 details how tasks were grouped in clinically important categories (e.g., prepare for delivery, basic interventions, ventilation, etc.). To pass the scenario, participants needed to complete all steps within each category (regardless of the order of the steps) before moving to the subsequent category. The digital simulation was programmed to follow the NRP algorithm. Any deviations from the algorithm resulted in a deterioration of the simulated infant's health. This deterioration may prompt participants to deviate further from the sequence expected by the digital simulator. Therefore, we decided to use pass/fail scoring to avoid this potential confounding interference. Descriptive statistics of the performance measures are reported as the mean and standard deviation (SD).

#### Predictor

Test time point is the predicting variable that represents which test the participant took. This categorical variable has four levels: (0: pretest; 1: immediate posttest; 2: posttest after 2 months; and 3: posttest after 5 months). In the following analysis, Level 0 (pretest) was regarded as the reference for comparison.

#### Moderator

*Mindset* was included as a moderator to measure the influence of time points on performance. Growth mindset reflects an individual's incremental intelligence, representing the belief that intelligence or ability can be improved through effort, whereas fixed mindset is an entity theory of intelligence, representing the belief that intelligence or ability cannot change, being fixed traits (26). The mindset moderator was calculated from the fixed and growth mindset items in the questionnaire. First, reversed coding was conducted on the two fixed mindset items so that they could constitute a latent trait continuum with the growth mindset. Then, we summed the reversed coded response scores of the two fixed mindset items (with possible range of 2–10) and the response scores of the two growth mindset items (with possible range of 2–10). Thus, the moderator was obtained as a continuum from low to high growth mindset, with values ranging from 4 to 20. We recorded the participants' initial survey responses on a 5-point Likert Scale (1: “*Strongly Disagree*,” 2: “*Disagree*,” 3: “*Neutral*,” 4: “*Agree*,” and 5: “*Strongly Agree*”). Then, we computed the *Mindset* continuum variable, with larger *Mindset* values indicating a higher endorsement of growth mindset.

### Statistical Analyses

General linear mixed model (GLMM) repeated measures with binary outcome variable were employed to analyze the longitudinal performance growth moderated by mindset. GLMMs are extensions of linear mixed models that handle response variables from a wider range of distributions (e.g., binary response variable) and that estimate the effects of both within-person and across-person variability through including both fixed and random effects. In the present study, the within-person variable is the *Predictor: Test time point with four levels*, the across-person variable is the *Moderator: Mindset*, and finally, the response variable is the binary performance score. The GLMM is shown in Equation (1):

(1) $$\begin{array}{l} ln\left(\frac{P\left(Y_{ij}=1\right)}{1-P\left(Y_{ij}=1\right)}\right)=\;\theta_{i}+\;\overset{K}{\underset{k=0}{\sum }}\beta_{k}X_{ijk}+\varepsilon_{ij}, \end{array}$$

where *Y*_*ij*_ represents the binary score (*Y*_*ij*_ = 0 or 1) of participant *I* (*i* = 1–50) at time point *j*. Specifically, *Y*_*ij*_ = 1 means that participant *i* passed the test at time point *j* and *Y*_*ij*_ = 0 means that participant *i* failed the test at time point *j* (*j* = 0: pretest; 1: immediate posttest; 2: posttest after 2 months; or 3: posttest after 5 months). In the current study, *j* = 0, 1, 2, 3, correspond to the pretest, immediate posttest, posttest after 2 months, and posttest after 5 months. Also, θ_*i*_ represents the overall latent ability of participant *i*, which follows a normal distribution with a mean of 0 and a standard deviation of 1, ε_*ij*_ is the error term with a normal distribution with a mean of 0 and a variance of π^2^/3, *X*_*ijk*_ are the predictor variable values for participant *i* at time point *j*, and the β_*k*_ parameters represent the *k*th predictor's fixed effect estimated by the model. Lastly, $\sum_{k=0}^{K}\beta_{k}X_{ijk}$ is the general fixed effect of *k* predictors.

In the present study, we fitted our data in three GLMMs with different predictor variables with or without interactions. Model 1 examines the effect of time points on task performance, which could illustrate participants' performance growth in the four subsequent tests, ignoring the moderation effect of mindset as shown in Equation (2). Model 2 in Equation (3) examines the effect of time points and mindset on an individual's test performance, which could reveal participants' changes in performance over time and the overall effect of mindset on performance. Model 3 in Equation (4) includes both the main effect of time points and mindset, and, in addition to Model 2, the interaction effect of time points and mindset. Model 3 helps us understand how mindset could potentially moderate participants' test performances on different tests with different levels of difficulty. *Participant ID* was introduced as a random effect in both models. The three models are summarized as follows:

Model 1
  (2) $$\begin{array}{l} ln\left(\frac{P\left(Y_{ij}=1\right)}{1-P\left(Y_{ij}=1\right)}\right)\\ =\theta_{i}+\;\beta_{Time1}X_{iTime1}+\;\beta_{Time2}X_{iTime2}\\ +\;\beta_{Time3}X_{iTime3}+\;\varepsilon_{ij}, \end{array}$$
Model 2
  (3) $$\begin{array}{l} ln\left(\frac{P\left(Y_{ij}=1\right)}{1-P\left(Y_{ij}=1\right)}\right)\\ =\theta_{i}+\;\beta_{Time1}X_{iTime1}+\;\beta_{Time2}X_{iTime2}\\ +\;\beta_{Time3}X_{iTime3}+\;\beta_{Mindset}X_{ijMindset}+\;\varepsilon_{ij} \end{array}$$
Model 3
  (4) $$\begin{array}{l} ln\left(\frac{P\left(Y_{ij}=1\right)}{1-P\left(Y_{ij}=1\right)}\right)\\ =\;\theta_{i}+\;\beta_{Time1}X_{iTime1}+\;\beta_{Time2}X_{iTime2}\\ +\;\beta_{Time3}X_{iTime3}+\;\beta_{Mindset}X_{ijMindset}\\ +\;\beta_{Time1:Mindset}X_{iTime1}+\;\beta_{Time2:Mindset}X_{iTime2}\\ +\;\beta_{Time3:Mindset}X_{iTime3}\;\varepsilon_{ij} \end{array}$$

The GLMM was performed on version 4.0.2 of the open statistical platform, *R* (35). The *glimmer* function in the *lme4* package (36) was used to estimate the effects of time point, mindset, and their interactions. Maximum Likelihood (ML) with Laplace approximation was used on parameter estimation.

## Result

### Descriptive Statistics

Table 1 presents the recruited HCPs' background information, time (in months) since their last NRP course, and experience in clinical neonatal care, video games, and educational games. The sample consisted of 27 registered nurses, 14 respiratory therapists, 6 doctors (including clinical fellows, residents, and clinical assistants), and 3 nurse practitioners. Participants were recruited from different educational backgrounds (Diploma: 3, Bachelor's Degree: 24, Master's Degree: 4, Medical Degree: 6, and After Degree: 4). HCPs had an average of 10.71 years (SD = 6.76) of experience in clinical neonatal care, an average time of 9.16 months (SD = 5.64) since their last NRP course, and an average of 7.45 years (SD = 9.87) of experience with video gaming. They spent about 6.26 h per month playing mobile/video games. Previous experience in video gaming would help facilitate HCPs' interaction with digital simulators. Only 20 HCPs reported that they had experience with educational games, whereas 30 HCPs were not exposed to educational games prior to this study.

**Table 1:** Descriptive statistics of participants' gender, time since last NRP course, highest level of education, registration, position, years of experience in clinical neonatal care, and experience of video games and educational games.

**Variable**	**Min**	**Max**	**Mean**	***SD***
Months of last NRP course	1	24	9.16	5.64
Years of neonatal care experience	0	30	10.71	6.76
Hours of videogame per month	0	60	6.26	14.40
Years of video game experience	0	35	7.46	9.87
Experience of educational games	Yes: 20			
	No: 30			
Highest levels of education	Diploma: 3			
	Bachelor's degree: 24			
	Master's degree: 4			
	Medical degree: 6			
	After degree: 4			
Registration	Registered nurses: 27			
	Respiratory therapists: 14			
	Doctors (Clinical Fellows/Residents/Clinical Assistants): 6			
	Nurse practitioners: 3			
Position	NNP/Fellow/Resident/CA: 10			
	Nurse: 26			
	Respiratory therapist: 13			
	Nursing/Medical/RT student: 1			
	Other			
Gender	Female: 44			
	Male: 6			

*Min, Minimum; Max, Maximum; SD, Standard Deviation*.

Table 2 displays the initial survey responses from the HCPs to the growth and fixed mindset items. For the items on growth mindset, most responses fall into the categories of “*Strongly Agree*” and “*Agree*.” For the items on fixed mindset, most responses fall into the categories of “*Strongly Disagree*” and “*Disagree*.” The responses to the mindset items demonstrate a general high level of growth mindset among the HCPs. After reverse coding the fixed mindset items, the growth mindset continuum was computed. The mean values of growth mindset (range: 4–20) and of the test scores (range: 0–1) are presented in Table 3. The 50 recruited HCPs highly endorsed a growth mindset, with a mean of 17.66 (SD = 1.64). On the performance assessments, HCPs obtained a mean score of 0.42 (SD = 0.5) on the pretest, which increased to 0.78 (SD = 0.42) on the immediate posttest. Their mean score on the posttest after 2 months decreased to 0.70 (SD = 0.46) and then increased to 0.80 (SD = 0.41) on the posttest after 5 months. Generally, participants increased their knowledge from the pretest to the subsequent posttests. From the immediate posttest, their scores decreased in the 2-month knowledge retention posttest but slightly increased on the 5-month transfer test.

**Table 2:** Participants' survey responses on growth and fixed mindset items.

**Variable**	**Strongly Disagree**	**Disagree**	**Neutral**	**Agree**	**Strongly Agree**	**Non-response**
Fixed Mindset1	18	31	1	0	0	0
Fixed Mindset2	17	32	1	0	0	0
Growth Mindset1	0	0	0	28	22	0
Growth Mindset2	0	0	0	22	28	0

**Table 3:** Descriptive statistics of participants' mindset and performance on the four tests.

**Variable**	**Min**	**Max**	**Mean**	***SD***
Mindset	15	20	17.66	1.64
Pretest	0	1	0.42	0.5
Posttest_Immediate	0	1	0.78	0.42
Posttest_2month	0	1	0.70	0.46
Posttest_5month	0	1	0.80	0.41

*Min, Minimum; Max, Maximum; SD, Standard Deviation*.

### Effect of Time Points Moderated by Mindset

**Figure 1:**
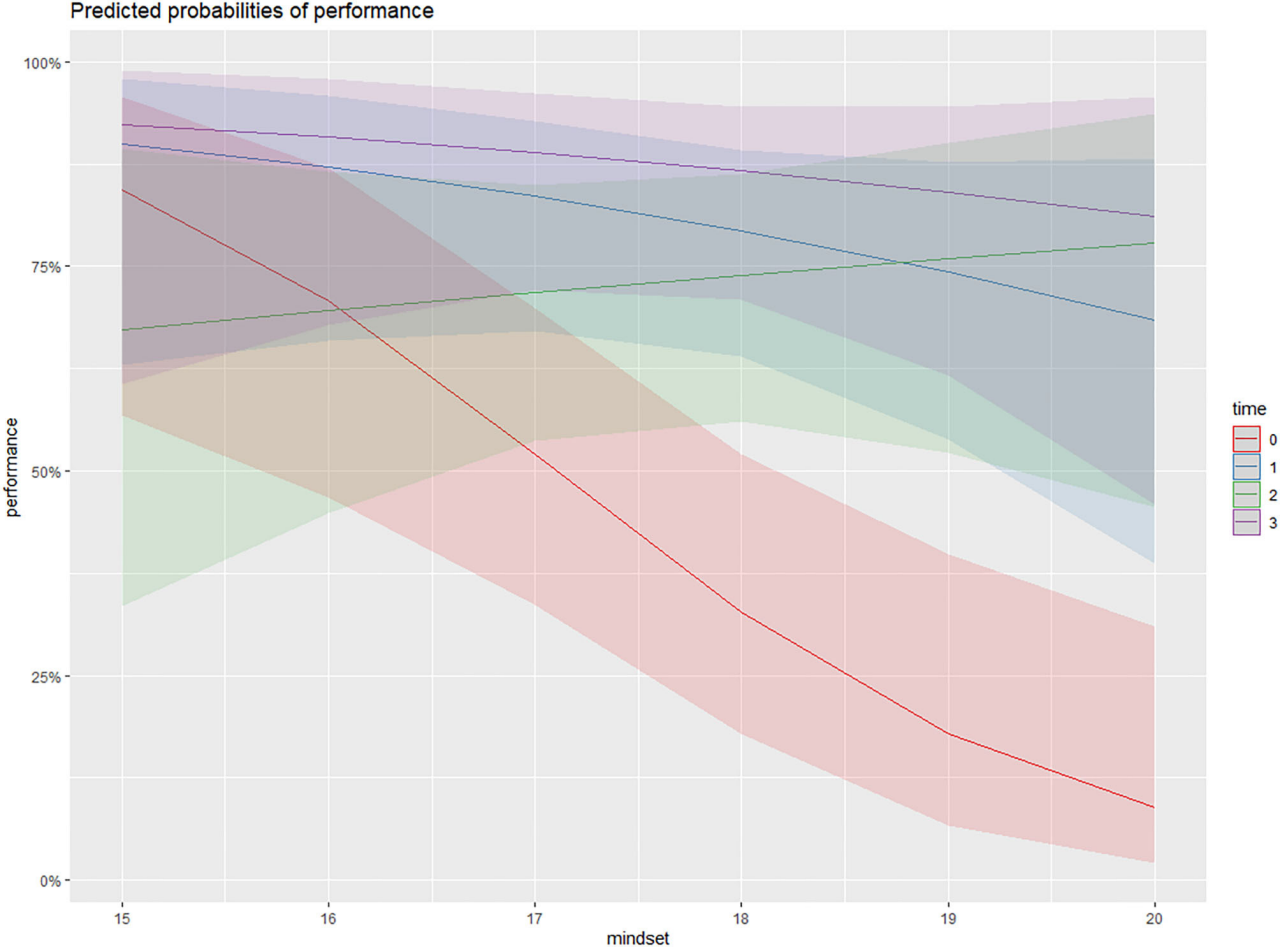
The interaction effect of Time * Mindset in Model 3. The *x*-axis represents participants' endorsement of growth mindset (range: 15–20). The *y*-axis represents the predicted response variable: *Test Performance* measured by the binary scores (range: 0–100%). The red, blue, green, and purple lines represent the trends of participants' predicted score when growth mindset increases (from 15 to 20) given a specific Test Time Point (time 0 = Pretest, time 1 = Immediate posttest, time 2 = 2-month posttest, and time 3 = 5-month posttest). The figure shows that although the three posttest scores are significantly higher than the pretest scores, there are no significant differences between the posttest scores. Only the interaction effect between time 2 * growth mindset is significantly positive (Est = 0.97, *p* < 0.01).

The effects of test time points and mindset on performance were probed using GLMM analyses. Results of the three models are presented in Table 4. Model 1 shows the main effect of time points. Findings revealed that the participants performed significantly better on the immediate posttest (*Est* = 1.88, *p* < 0.05), the posttest after 2 months (*Est* = 1.36, *p* < 0.05), and the posttest after 5 months (*Est* = 2.01, *p* < 0.05), compared with the pretest. Model 2 reveals the main effect of time points and mindset on the overall performance on the tests; similarly, participants performed significantly better on the immediate posttest (*Est* = 1.89, *p* < 0.05), the posttest after 2 months (*Est* = 1.35, *p* < 0.05), and the posttest after 5 months (*Est* = 2.01, *p* < 0.05), after taking the pretest and the simulation-based neonatal resuscitation training. To further examine pairwise differences between the four tests, a *post-hoc* analysis was performed. Table 4 and Figure 1 demonstrate that, although the three posttest scores are significantly higher than the pretest scores, there are no significant differences between the scores of the posttests. Thus, participants mastered and retained the neonatal resuscitation knowledge after the digital simulation training, despite some performance fluctuations from time point to time point. In addition, more of a growth mindset has a significant negative main effect on the immediate posttest performance (*Est* = −0.30, *p* < 0.05). The results indicate that participants are more likely to score higher on the simulation-based assessments, if they show lower endorsement of growth mindset (or higher endorsement of a fixed mindset), regardless of the effect of time.

**Table 4:** Summary of the three GLMM models.

	**Model1**			**Model2**			**Model3**		
**Coefficients**	**Est**	**SE**	***p***	**Est**	**SE**	***p***	**Est**	**SE**	***p***
(Intercept)	−0.39	0.34	0.25	**4.86**	2.33	0.04	**13.76**	4.42	0.00
Time1_Immediate Posttest	**1.88**	0.52	0.00	**1.89**	0.48	0.00	−7.16	5.93	0.23
Time2_Posttest after 2 months	**1.36**	0.50	0.01	**1.35**	0.47	0.00	**−15.64**	5.71	0.01
Time3_Posttest after 5 months	**2.01**	0.56	0.00	**2.01**	0.52	0.00	−8.19	6.37	0.20
Mindset				**−0.30**	0.13	0.02	**−0.81**	0.25	0.00
Time1: Mindset							0.52	0.34	0.12
Time2: Mindset							**0.97**	0.33	0.00
Time3: Mindset							0.59	0.36	0.10
AIC	219.8			216.8			213.4		
BIC	235.8			236.0			242.3		
Log likelihood	−104.9			−102.4			−97.7		
Deviance	209.8			204.8			195.4		
Degrees of freedom residual	178			177			174		

*Est, Estimate; SE, Standard Error; AIC, Akaike Information Criterion; BIC, Bayesian Information Criterion; the values marked in bold represent significant coefficient estimates*.

**Figure 2:**
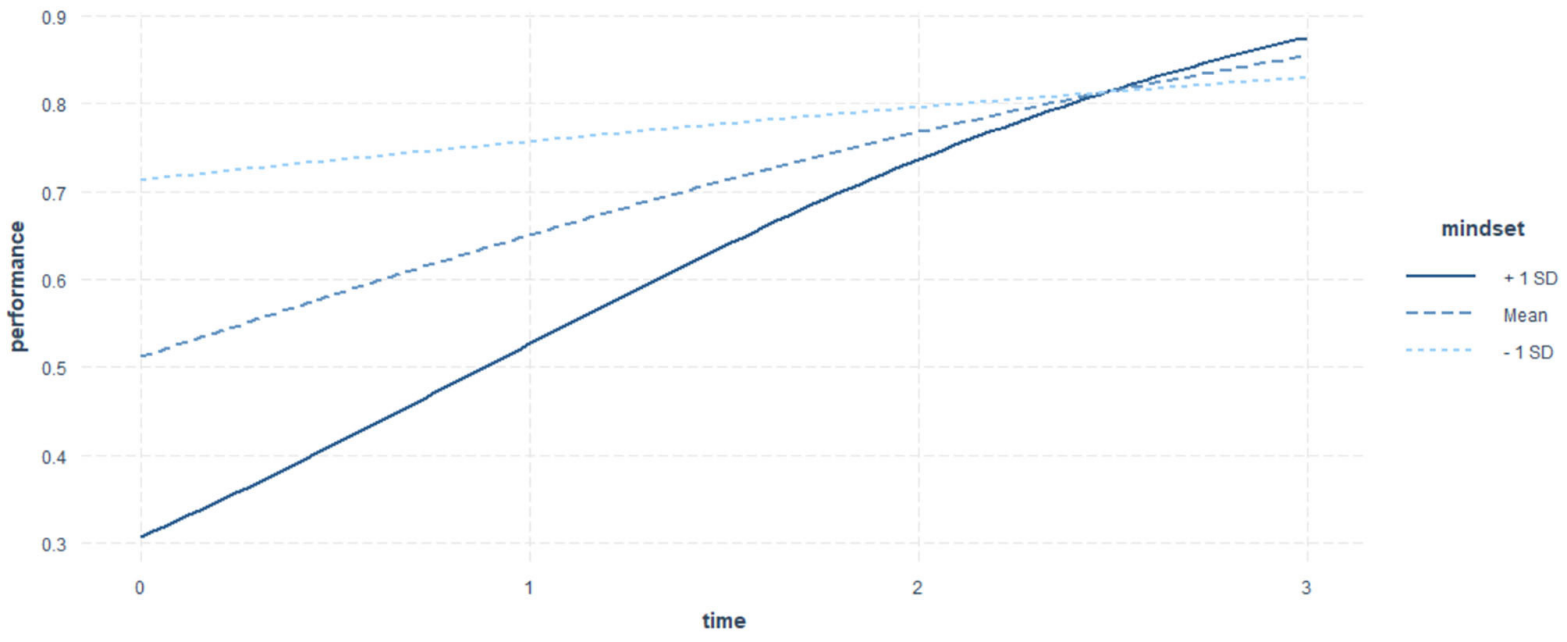
The effect of mindset over test time point. The *x*-axis represents the predictor Test Time Point with three levels: 0 = Pretest, 1 = Immediate posttest, 2 = 2-month posttest, and 3 = 5-month posttest. The *y*-axis represents the predicted response variable: Test Performance measured by the binary scores (0 or 1). The growth mindset moderator is represented by the three curves, where the bolded full curve represents the participant with growth mindset at the value of Mean + 1 SD (17.66 + 1.64), the dashed line in dark blue represents the participant with growth mindset at the value of Mean (17.66), and the dashed line in light blue represents the participants with growth mindset at the value of Mean −1 SD (17.66 – 1.64). The trajectories of the three curves demonstrate the changes of test performance with different levels of growth mindset. Participants with the highest growth mindset (Mean + 1 SD) demonstrate highest improvements from the pretest to the last posttest. Although they are predicted to perform the poorest on the pretest, GLMM shows that they are likely to surpass the participants with average and lower growth mindset after the 2-month posttest and perform best among all participants. In contrast, participants with the lowest growth mindset (mean −1 SD) showed the least improvements in knowledge retention. Finally, participants with an average endorsement of growth mindset show a moderate improvement on the test performance over time. The figure shows that participants with higher levels of growth mindset generally show greater improvements over time (i.e., from the pretest, to the immediate, to the 2-months, and to the 5-month posttests).

Model 3 examines the effects of time, mindset, and their interaction on performance. Results show that, generally, mindset has a positive effect on every performance test. However, only the interaction effect *Time2* * *Mindset* (*Est* = 0.97, *p* < 0.05) was significant, suggesting that growth mindset has a positive effect on performance of the posttest after 2 months compared with other posttests. Figure 2 illustrates the performance growth trends on each time point shaped by mindset. In the pretest, the stronger the growth mindset, the lower the performance score predicted by the model. In the immediate posttest and the 5-month posttest, growth mindset had a minor negative effect on performance. In contrast, a participant was more likely to achieve a higher performance on the 2-month posttest when endorsing a stronger growth mindset.

To visualize the effect of mindset on test performance over time, we plotted the interaction effect *Time* * *Mindset* on performance in Figure 2. The three lines represent the growth curves of participants with different levels of growth mindset estimated by the GLMM. Participants with mean growth mindset levels represented by the bold dashed line are predicted to start with a medium performance on the pretest and show a stable increase in scores at a medium speed. On the other hand, participants endorsing lower levels of growth mindset (mean growth mindset minus one standard deviation) are predicted to have the highest score on the pretest but improve their performance at the lowest speed. Finally, participants who endorse higher levels of a growth mindset (mean growth mindset plus one standard deviation) represented by the solid dashed line started with the lowest scores on the pretest before the simulation-based training but showed the largest growth on performance over time, surpassing the other two categories after the first delayed posttest and achieving the highest performance on the last test.

## Discussion

In this study, we investigated whether HCPs benefited from the RETAIN digital and table-top simulation-based neonatal resuscitation training. Specifically, we explored whether HCPs improved their performance on longitudinal tests using the RETAIN digital simulator and transferred their knowledge to a new medium, the table-top simulator. Further, we examined whether the mindset moderator influenced HCPs' task performance at different time points. Results show that participants performed significantly better on the digital simulation tests as well as the table-top simulation test compared with the pretest. Moreover, although there were some fluctuations in performance (i.e., participants' mean scores on the 2-month posttest decreased compared with the scores on the immediate posttest but increased again on the 5-month posttest), there were no significant differences in the performance of the three posttests. The general best performance on the last posttest indicates a possible knowledge transfer. Overall, participants experienced knowledge gain, retention, and transfer after using the digital simulation-based training.

Previous studies on mindset sampled mostly children or students in different grade levels to observe the effects of mindset on improving academic achievement (27–34). Few studies examined the long-term impact of growth mindset on professional training, knowledge retention, and transfer of skilled specialized personnel in different areas. Some of the previous studies revealed that growth mindset interventions could effectively foster student learning beliefs and thus prepare them for sizable improvements of learning outcomes (31, 33). Others argued that the facilitative effects of growth mindset intervention are too small given the large amount of time and labor investment (30). This study presents a longitudinal investigation of the impact of growth mindset on the performance of highly skilled personnel. The results from the present study indicate that endorsing more of a growth mindset has an overall negative effect on performance when ignoring the fixed effect of within-subject predictor time points. However, growth mindset has a positive effect on performance on the second time point (2-month posttest) after introducing the interaction term of time^*^mindset. Furthermore, the interaction effect is not significant on the immediate posttest but becomes significant on the first delayed posttest (i.e., after 2 months). This effect did not reach significance on the transfer test (5-month posttest), likely because of the small sample size and the different nature of the posttest (table-top simulator, instead of a digital simulator as was the case with the other tests). Moreover, the model fit line shows that participants with higher endorsement of a growth mindset demonstrated not only better performance on the last two posttests but also larger improvements during the 5 months. Conversely, participants endorsing more of a fixed mindset show a slower progression in performance, likely because they did not believe that practice could improve their performance. Participants endorsing more of a growth mindset started with slightly lower scores on the pretest but improved their performance at a faster speed and achieved a higher performance in the long term. Therefore, the results of this study suggest that endorsing a growth mindset could facilitate long-term technology-based learning.

## Conclusions, Limitations, and Implications

The current study empirically examines the effectiveness of a digital neonatal resuscitation simulation training program through a series of longitudinal tests and further inspects whether and how mindset potentially moderates the long-term outcomes of technology-based learning. Results show that HCPs successfully mastered neonatal resuscitation knowledge after the training and practice using digital simulators, improved their test performance on the digital simulator, retained their knowledge as shown by the 2-month delayed posttest, and transferred their knowledge to the table-top simulator after 5 months. Growth mindset did not moderate the gain in performance from the pretest to the immediate posttest. However, on the 2-month delayed posttest, a growth mindset was significantly associated with better performance. Limitations of this study include the small sample size and skewed gender distribution toward females, which is mainly attributed to the highly specialized profession and the predominance of females in the intensive neonatal care center. Future studies can be conducted in other domains to understand the impact of mindset on knowledge acquisition and retention of skilled professionals in various fields. Findings of the present study demonstrate the potential of digital simulators on neonatal resuscitation training and inspire future research on implementing growth mindset interventions along with technology-enhanced learning and assessment to promote neonatal resuscitation knowledge gain, retention, and transfer.

## Data Availability Statement

The raw data supporting the conclusions of this article will be made available by the authors, without undue reservation.

## Ethics Statement

The studies involving human participants were reviewed and approved by the present study was approved by the Human Research Ethics Board at the University of Alberta (Pro00064234). The patients/participants provided their written informed consent to participate in this study.

## Author Contributions

GS, MC, SG, and CL conceptualized and designed the study, drafted the initial manuscript, and critically reviewed and revised the manuscript. All authors approved the final manuscript as submitted and agree to be accountable for all aspects of the work.

## Conflict of Interest

GS has registered the RETAIN table-top simulator (Tech ID 2017083) and the RETAIN digital simulator under Canadian copyright (Tech ID—2017086). The remaining authors declare that the research was conducted in the absence of any commercial or financial relationships that could be construed as a potential conflict of interest.

## Abbreviations

Est: Estimate
GLMM: Generalized Linear Mixed Model
HALO: High Acuity Low Occurrence
HCP: Healthcare Provider
ML: Maximum Likelihood
NRP: Neonatal Resuscitation Program
RETAIN: REsuscitation TrAINing for healthcare providers
SD: Standard Deviation
SE: Standard Error
AIC: Akaike Information Criterion
BIC: Bayesian Information Criterion.

## References

[1] Mitchell A Niday P Boulton J Chance G Dulberg C. A prospective clinical audit of neonatal resuscitation practices in Canada. Adv Neonatal Care. (2002) 6:316–26. 10.1053/adnc.2002.36831 12881944

[2] Perlman Jeffrey M Jonathan W John K Wyckoff Myra H Khalid A Ruth G. Part 7: neonatal resuscitation. Circulation. (2015) 132:S204–41. 10.1161/CIR.0000000000000276 26472855

[3] Wyckoff MH Aziz K Escobedo MB Kapadia VS Kattwinkel J Perlman JM. Part 13: neonatal resuscitation: 2015 American Heart Association Guidelines Update for Cardiopulmonary Resuscitation and Emergency Cardiovascular Care. Circulation. (2015) 132(18 Suppl 2):543. 10.1161/CIR.0000000000000267 26473001

[4] Holtzman D Cloyd J Sammann A Tendick F O'Sullivan P Ascher N. QS44. Surgical mentorship and operating room experience for preclerkship medical students. J Surg Res. (2008) 144:287. 10.1016/j.jss.2007.12.282 18707660

[5] Cutumisu M Brown MRG Fray C Schmölzer GM. Growth mindset moderates the effect of the neonatal resuscitation program on performance in a computer-based game training simulation. Front Pediatr. (2018) 6:195. 10.3389/fped.2018.00195 30023355 PMC6039560

[6] Hexter AT O'Dowd-Booth C Hunter A. Factors that influence medical student learning in the operating room. Med Teach. (2019) 41:555–60. 10.1080/0142159X.2018.1504163 30253684

[7] Lee FQH Chua WJ Cheong CWS Tay KT Hian EKY Chin AMC. A systematic scoping review of ethical issues in mentoring in surgery. J Med Educ Curric Dev. (2019) 6:2382120519888915. 10.1177/2382120519888915 31903425 PMC6923696

[8] Williams AL Lasky RE Dannemiller JL Andrei AM Thomas EJ. Teamwork behaviours and errors during neonatal resuscitation. BMJ Qual Saf. (2010) 19:60–4. 10.1136/qshc.2007.025320 20172885

[9] Yamada NK Fuerch JH Halamek LP. Impact of standardized communication techniques on errors during simulated neonatal resuscitation. Am J Perinatol. (2016) 33:385–92. 10.1055/s-0035-1565997 26485251

[10] Yamada NK Yaeger KA Halamek LP. Analysis and classification of errors made by teams during neonatal resuscitation. Resuscitation. (2015) 96:109–13. 10.1016/j.resuscitation.2015.07.048 26282500

[11] Garvey AA Dempsey EM. Simulation in neonatal resuscitation. Front Pediatr. (2020) 8:59. 10.3389/fped.2020.00059 32158737 PMC7052260

[12] Campbell DM Barozzino T Farrugia M Sgro M. High-fidelity simulation in neonatal resuscitation. Paediatr Child Health. (2009) 14:19–23. 10.1093/pch/14.1.19 19436459 PMC2661330

[13] Cutumisu M Patel SD Brown MRG Fray C von Hauff P Jeffery T. RETAIN: A board game that improves neonatal resuscitation knowledge retention. Front Pediatr. (2019) 7:13. 10.3389/fped.2019.00013 30766862 PMC6365420

[14] Ghoman SK Schmölzer GM. The RETAIN simulation-based serious game-a review of the literature. Healthcare. (2020) 8:3. 10.3390/healthcare8010003 31877882 PMC7151097

[15] Ghoman SK Cutumisu M Schmölzer GM. Simulation-based summative assessment of neonatal resuscitation providers using the RETAIN serious board Game-A pilot study. Front Pediatr. (2020) 8:14. 10.3389/fped.2020.00014 32083041 PMC7006050

[16] Halamek LP Kaegi DM Gaba DM Sowb YA Smith BC Smith BE. Time for a new paradigm in pediatric medical education: teaching neonatal resuscitation in a simulated delivery room environment. Pediatrics. (2000) 106:e45. 10.1542/peds.106.4.e45 11015540

[17] Palmer E Labant AL Edwards TF Boothby J. A collaborative partnership for improving newborn safety: using simulation for neonatal resuscitation training. J Contin Educ Nurs. (2019) 50:319–24. 10.3928/00220124-20190612-07 31233606

[18] Alinier G Hunt B Gordon R Harwood C. Effectiveness of intermediate-fidelity simulation training technology in undergraduate nursing education. J Adv Nurs. (2006) 54:359–69. 10.1111/j.1365-2648.2006.03810.x 16629920

[19] Laschinger S Medves J Pulling C McGraw DR Waytuck B Harrison MB. Effectiveness of simulation on health profession students' knowledge, skills, confidence and satisfaction. Int J Evid Based Healthcare. (2008) 6:278–302. 10.1097/01258363-200809000-00002 21631826

[20] Huang J Tang Y Tang J Shi J Wang H Xiong T. Educational efficacy of high-fidelity simulation in neonatal resuscitation training: a systematic review and meta-analysis. BMC Med Educ. (2019) 19:323. 10.1186/s12909-019-1763-z 31464614 PMC6716944

[21] Andreatta PB Hillard M Krain LP. The impact of stress factors in simulation-based laparoscopic training. Surgery. (2010) 147:631–9. 10.1016/j.surg.2009.10.071 20414972

[22] Demaria S Jr Bryson EO Mooney TJ Silverstein JH Reich DL Bodian C. Adding emotional stressors to training in simulated cardiopulmonary arrest enhances participant performance. Med Educ. (2010) 44:1006–15. 10.1111/j.1365-2923.2010.03775.x 20880370

[23] Fraser K McLaughlin K. Temporal pattern of emotions and cognitive load during simulation training and debriefing. Med Teach. (2019) 41:184–9. 10.1080/0142159X.2018.1459531 29687734

[24] Ravindra P Fitzgerald JE Bhangu A Maxwell-Armstrong CA. Quantifying factors influencing operating theater teaching, participation, and learning opportunities for medical students in surgery. J Surg Educ. (2013) 70:495–501. 10.1016/j.jsurg.2013.02.011 23725937

[25] Patel V Aggarwal R Osinibi E Taylor D Arora S Darzi A. Operating room introduction for the novice. Am J Surg. (2012) 203:266–75. 10.1016/j.amjsurg.2011.03.003 21703594

[26] Dweck CS. Self-Theories: Their Role in Motivation, Personality, and Development. New York NY: Psychology Press (2000).

[27] Bahník Š Vranka MA. Personality and individual differences. Pers Indiv Differ. (1980) 117:139–43. 10.1016/j.paid.2017.05.046

[28] Corradi D Nicolaï J Levrau F. Growth mindset and its predictive validity-do migration background and academic validation matter? High Educ. (2019) 77:491–504. 10.1007/s10734-018-0286-6

[29] Li Y Bates TC. Testing the association of growth mindset and grades across a challenging transition: is growth mindset associated with grades? Intelligence (Norwood). (2020) 81:101471. 10.1016/j.intell.2020.101471

[30] Sisk VF Burgoyne AP Sun J Butler JL Macnamara BN. To what extent and under which circumstances are growth mind-sets important to academic achievement? Two Meta-Analyses. Psychol Sci. (2018) 29:549–71. 10.1177/0956797617739704 29505339

[31] Sobral DT. Medical students' mindset for reflective learning: a revalidation study of the reflection-in-learning scale. Adv Health Sci Educ. (2005) 10:303–14. 10.1007/s10459-005-8239-0 16362619

[32] Tang X Wang M Guo J Salmela-Aro K. Building Grit: the longitudinal pathways between mindset, commitment, grit, and academic outcomes. J Youth Adolesc. (2019) 48:850–63. 10.1007/s10964-019-00998-0 30788767

[33] Yeager DS Hanselman P Walton GM Murray JS Crosnoe R Muller C. A national experiment reveals where a growth mindset improves achievement. Nature (London). (2019) 573:364–9. 10.1038/s41586-019-1466-y 31391586 PMC6786290

[34] Zhang J Kuusisto E Tirri K. How teachers' and students' mindsets in learning have been studied: research findings on mindset and academic achievement. Psychology. (2017) 8:1363–77. 10.4236/psych.2017.89089

[35] RC Team. R Core Team. R: A Language and Environment for Statistical Computing (2015).

[36] Bates D Maechler M Bolker B Walker S Christensen RHB Singmann H. Package 'lme4' Version. (2018) 1:17.

