# Supplementary material for: Mindset Moderates Healthcare Providers' Longitudinal Performance in a Digital Neonatal Resuscitation Simulator
Source: Front Pediatr. 2021 Feb 16;8:594690. doi: 10.3389/fped.2020.594690 (PMC7921319; doi:10.3389/fped.2020.594690)
Supplement: Supplementary file 2 [file Data_Sheet_2.PDF]

Supplementary Material

1 Appendix 1

1.1 Pre-test, post-test, and 2-month post-test scenario

1. Prepare for the delivery. Assign roles, check equipment, set ventilation device, gather supplies, call for assistance, and don personal protective equipment. Review the case history (term baby, clear fluid, and fetal bradycardia for the last 3 minutes). Discuss the plan for cord management with the obstetrician.
2. The baby has been born. Immediate cord clamping due to the baby being apneic and having poor muscle tone.
3. Complete initial assessment. Visual assessment reveals the baby has no muscle tone. Breathing and airway assessment reveals the baby is apneic. After auscultation, the heart rate is 40 beats per minute.
4. Complete basic interventions. Dry and maintain temperature; tactile stimulation, measure oxygen saturation, measure heart rate, and measure temperature. The heart rate is 40 beats per minute.
5. Initiate ventilation. After initiating positive pressure ventilation, heart rate is 40 beats per minute after 15 seconds, with no chest rise. Begin ventilation corrective steps. Adjust mask and reposition airway. The heart rate is 40 beats per minute. Suction the airway and open the mouth. The heart rate is 40 beats per minute. Adjust ventilation pressure. Heart rate is 40 beats per minute. Increase inspired oxygen concentration (or earlier).
6. Establish an alternative airway and confirm correct tube placement. The first attempt to establish an alternative airway is unsuccessful. Remove device, resume positive pressure ventilation, and repeat insertion. The second attempt to establish an alternative airway is successful. The heart rate is 40 beats per minute, and there is chest rise, misting of the tube, and end-tidal CO_2. Continue positive pressure ventilation.
7. Initiate cardiovascular interventions. Increase oxygen concentration to 100% if not already. Give chest compressions. After 60 seconds of chest compressions, the heart rate is 70 beats per minute. Stop chest compressions and continue positive pressure ventilation. Adjust inspired oxygen concentration to maintain oxygen saturation according to local hospital policy.
8. Post-resuscitation. Ten minutes after birth, the baby is stabilized. The heart rate is 141 beats per minute and the oxygen saturation is 90%. Prepare to admit the baby to the NICU. Debrief.

1.2 5-month post-test scenario

1. Prepare for the delivery. Assign roles, check equipment, set ventilation device, gather supplies, call for assistance, and don personal protective equipment. Review the case history. Discuss the plan for cord management with the obstetrician.
2. The baby has been born. Immediate cord clamping due to the baby being apneic and having poor muscle tone. Visual assessment reveals thick meconium.

3. Complete initial assessment. Visual assessment reveals the baby has no muscle tone. Breathing and airway assessment reveals the baby is apneic, and the airways are blocked with meconium. After auscultation, the heart rate is 34 beats per minute.
4. Complete basic interventions. Dry and maintain temperature; tactile stimulation (optional), suction airway, measure oxygen saturation, measure heart rate, and measure temperature. The heart rate is 37 beats per minute.
5. Initiate ventilation. After initiating positive pressure ventilation, heart rate is 36 beats per minute after 15 seconds, with no chest rise. Begin ventilation corrective steps. Adjust mask and reposition airway. The heart rate is 34 beats per minute. Suction the airway and open the mouth. The heart rate is 35 beats per minute. Adjust ventilation pressure. Heart rate is 33 beats per minute. Increase inspired oxygen concentration (or earlier).
6. Establish an alternative airway and confirm correct tube placement. The first attempt to establish an alternative airway is successful. The heart rate is 42 beats per minute, and there is chest rise, misting of the tube, and end-tidal CO_2. Continue positive pressure ventilation.
7. Initiate cardiovascular interventions. Increase oxygen concentration to 100% if not already. Give chest compressions. After 60 seconds of chest compressions, the heart rate is 43 beats per minute.
8. Establish vascular access. Vascular access is successfully obtained. After 60 seconds of chest compressions, the heart rate is 49 beats per minute.
9. Administer medication. Continue chest compressions and administer the first dose of epinephrine. After 60 seconds of chest compressions and the first dose of epinephrine, the heart rate is 89 beats per minute. Stop chest compressions and continue positive pressure ventilation. Adjust inspired oxygen concentration to maintain oxygen saturation according to local hospital policy.
10. Post-resuscitation. Ten minutes after birth, the baby is stabilized. The heart rate is 141 beats per minute and the oxygen saturation is 97%. Prepare to admit the baby to the NICU. Debrief.

2 Appendix 2 The Survey Instrument

Abbreviated name	Full name	Units
Participant_ID	Participant_ID	1001 to 1050
Gender	Which best describes your gender identity?	1 = Female<br>2 = Male<br>3 = Other not listed
Months_NRP	Time (in months) since your last NRP course	Open answer (months)
Education	What is the highest level of education you have completed?	1 = Diploma<br>2 = Bachelor's<br>3 = After Degree<br>4 = Master's<br>5 = MD<br>6 = PhD<br>7 = Other
Registration	What is your registration(s), if any?	1 = MD (Neonatologist)<br>2 = Neonatal Nurse Practitioner<br>3 = MD (Fellow/Resident/Clinical Assistant)<br>4 = Registered Nurse<br>5 = Respiratory Therapist<br>6 = Other
Position	What is your current position?	1 = Physician<br>2 = NNP/Fellow/Resident/CA<br>3 = Nurse<br>4 = Respiratory Therapist<br>5 = Nursing/Medical/RT student<br>6 = Other
Years_Neonatal	How many years of experience in clinical neonatal care do you have?	Open answer (years)
Hours_Videogame	How many hours do you spend playing mobile/video games in a typical month?	Open answer
Years_Videogame_Experience	How many overall years of videogaming experience do you have?	Open answer
Experience_Educationalgames	Do you have any previous experience with educational video games?	1 = Yes<br>2 = No

Can’t_Change	You can't really do much to change how good you are at your job.	1 = Strongly Disagree<br>2 = Disagree<br>3 = Neutral<br>4 = Agree<br>5 = Strongly Agree
Can_Learn_Can’t_Change	You can learn new things, but you cannot really change how good you are at your job.	1 = Strongly Disagree<br>2 = Disagree<br>3 = Neutral<br>4 = Agree<br>5 = Strongly Agree
Can_Change	You can always change how good you are at your job.	1 = Strongly Disagree<br>2 = Disagree<br>3 = Neutral<br>4 = Agree<br>5 = Strongly Agree
Can_Change_Practice	You can get better at your job with practice.	1 = Strongly Disagree<br>2 = Disagree<br>3 = Neutral<br>4 = Agree<br>5 = Strongly Agree
